# Supplementary material for: Removal of V(V) From Solution Using a Silica-Supported Primary Amine Resin: Batch Studies, Experimental Analysis, and Mathematical Modeling
Source: Molecules. 2020 Mar 23;25(6):1448. doi: 10.3390/molecules25061448 (PMC7145307; doi:10.3390/molecules25061448)
Supplement: Supplementary file 1 [file molecules-25-01448-s001.pdf]

molecules

Supplementary Materials: Removal of V(V) From Solution Using a Silica-Supported Primary Amine Resin: Batch Studies, Experimental Analysis, and Mathematical Modeling

Xi Huang [1,*], Zhenxiong Ye [2], Lifeng Chen [3], Xujie Chen [4], Caocong Liu [4], Yuan Yin [1], Xinpeng Wang [4,*] and Yuezhou Wei [3,4].

[1] Advanced Nuclear Energy Research Group, College of Physics and Optoelectronic Engineering, Shenzhen University, Shenzhen 518060, China; yinyuan@szu.edu.cn

[2] College of Chemistry and Chemical Engineering, Guangxi University, Nanning 530004, China; 1715308005@st.gxu.edu.cn

[3] School of Nuclear Science and Engineering, Shanghai Jiao Tong University, 800 Dong Chuan Road, Shanghai 200240, China; chenlf@sjtu.edu.cn (L.C.); yzwei@sjtu.edu.cn (Y.W.)

[4] School of Resources, Environment and Materials, Guangxi Key Laboratory of Processing for Non-ferrous Metallic and Featured Materials, Guangxi University, Nanning 530004, China; 18894826954@163.com (X.C.); liucaocong@163.com (C.L.)

* Correspondence: xi.huang@szu.edu.cn (X.H.); wangxinpeng@gxu.edu.cn (X.W.); Tel.:+86-0755-26905882 (X.H.); +86-0771-3224332(X.W.); Fax: +86-0755-26538580 (X.H.); +86-0771-3224332 (X.W.)

Received: 16 February 2020; Accepted: 20 March 2020; Published: date

Abstract: Every year, a large quantity of vanadium-containing wastewater is discharged from industrial factories, resulting in severe environmental problems. In particular, V(V) is recognized as a potentially hazardous contaminant due to its high mobility and toxicity, and it has received considerable attention. In this study, a silica-supported primary amine resin (SiPAR) was prepared by in-situ polymerization, and the V(V) adsorption from the solution was examined. The as-prepared resin exhibited fast adsorption kinetics, and it could attain an equilibrium within 90 min for the V(V) solution concentration of 100 mg/L at an optimum pH of 4, whereas the commercial D302 resin required a treatment time of more than 3 h under the same conditions. Furthermore, the maximum adsorption capacity of the resin under optimum conditions for V(V) was calculated to be 70.57 mg/g. In addition, the kinetics and isotherm data were satisfactorily elucidated with the pseudo-second-order kinetics and Redlich–Peterson models, respectively. The silica-based resin exhibited an excellent selectivity for V(V), and the removal efficiency exceeded 97% in the presence of competitive anions at 100 mmol/L concentrations. The film mass-transfer coefficient (k_f) and V(V) pore diffusivity (D_p) onto the resins were estimated by mathematical modeling. In summary, this study provided a potential adsorbent for the efficient removal of V(V) from wastewater.

Keywords: pentavalent vanadium; removal; silica-supported resin; mathematical modeling

Acid-base titration for the determination of functional groups

2.0 g of SiPAR was placed in a dry triangular bottle, and then mixed with 50 mL of HCl standard solution with a concentration of 0.1018 mol/L. The mixture was stirred for 2 h at room temperature. Then, 25 mL of supernatant was taken and added to a beaker, which was used for titration. Finally, the residual concentration of HCl solution was analyzed by an automatic potentiometric titrator (Metrohm 916, Switzerland). The total exchange capacity of the SiPAR was calculated as follows:

$$q_t = \frac{50C_H - 2C_{OH}V_{OH}}{m}$$

where q_t (mmol/g) denotes the total exchange capacity, m (g) is the mass of the SiPAR, C_H and C_{OH} (mmol/mL) are the concentration of HCl and NaOH used in the titration, respectively, and V_{OH} (mL) denotes the volume of NaOH used for the titration of 25 mL of supernatant.

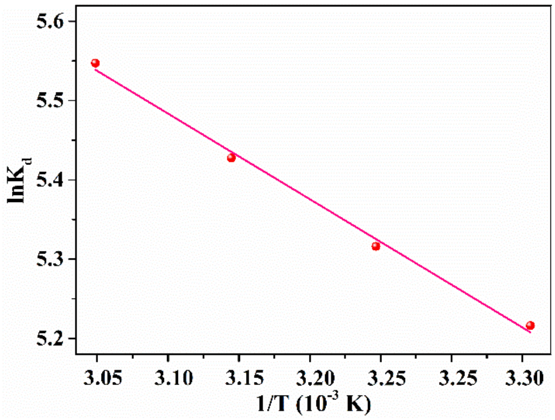

Figure S1. Plot of $\ln K_d$ versus 1/T for the V(V) adsorption onto SiPAR.

Table S1. Thermodynamic parameters for V(V) adsorption onto SiPAR.

Δ*G* (kJ/mol)				Δ*H* (kJ/mol)	Δ*S* (J/(mol K))
298K	308 K	318 K	328 K		
- 12.91	-13.64	- 14.37	-15.11	8.96	73.38
